# Curcumin and Intestinal Oxidative Stress of Pigs With Intrauterine Growth Retardation: A Review

**DOI:** 10.3389/fnut.2022.847673

**Published:** 2022-04-28

**Authors:** Xiaopeng Tang, Kangning Xiong, Teketay Wassie, Xin Wu

**Affiliations:** ^1^State Engineering Technology Institute for Karst Desertfication Control, School of Karst Science, Guizhou Normal University, Guiyang, China; ^2^CAS Key Laboratory of Agro-Ecological Processes in Subtropical Region, National Engineering Laboratory for Pollution Control and Waste Utilization in Livestock and Poultry Production, Hunan Provincial Engineering Research Center for Healthy Livestock and Poultry Production, Institute of Subtropical Agriculture, Chinese Academy of Sciences, Changsha, China; ^3^Laboratory of Nutrient Resources and Synthetic Biology, Tianjin Institute of Industrial Biotechnology, Chinese Academy of Sciences, Tianjin, China

**Keywords:** curcumin, intestinal health, intrauterine growth retardation, oxidative stress, pigs

## Abstract

Intrauterine growth restriction (IUGR) refers to the slow growth and development of a mammalian embryo/fetus or fetal organs during pregnancy, which is popular in swine production and causes considerable economic losses. Nutritional strategies have been reported to improve the health status and growth performance of IUGR piglets, among which dietary curcumin supplementation is an efficient alternative. Curcumin is a natural lipophilic polyphenol derived from the rhizome of *Curcuma longa* with many biological activities. It has been demonstrated that curcumin promotes intestinal development and alleviates intestinal oxidative damage. However, due to its low bioavailability caused by poor solubility, chemical instability, and rapid degradation, the application of curcumin in animal production is rare. In this manuscript, the structural-activity relationship to enhance the bioavailability, and the nutritional effects of curcumin on intestinal health from the aspect of protecting piglets from IUGR associated intestinal oxidative damage were summarized to provide new insight into the application of curcumin in animal production.

## Introduction

Intrauterine growth restriction (IUGR), is defined as the slow growth and development of a mammalian embryo/fetus or fetal organs during pregnancy, which has become a difficult problem in human medicine and animal husbandry (1, 2). Pig is a kind of mammal animal with multiple pregnancies, it has a high incidence of IUGR, which would not only reduce the survival rate of the newborn piglets but also affect the growth and development and health status of piglets in a longer period after birth (3–5). Therefore, it is of great significance for the economic benefits of pig production to improve the health status of IUGR piglets, improve their survival rate and growth performance through nutritional strategies. Meanwhile, due to the high similarities between pigs and humans in anatomy, physiology, and nutrient metabolism, the IUGR pigs can be used as an ideal animal model to study human diseases (6, 7).

The intestinal tract is the direct place for the communication between the internal environment and the external environment and is an important defense line for animals to maintain the homeostasis of the internal environment (8–10). Optimum intestinal health is of prime importance to animal growth as well as animal health. Previous studies have revealed that IUGR caused a significant negative effect on the growth and development of the gastrointestinal tract of piglets, manifested by the decreased intestinal length and weight, decreased villus height (VH) and increased crypt depth (CD), increased apoptosis of intestinal epithelial cells, and increased oxidative damage (11–14). The impaired development of the gastrointestinal tract is likely to be the main reason for retard growth and the poor health status of IUGR piglets (6, 15–17). The growing body of evidence has shown that the health status and growth performance of IUGR piglets can be improved through nutritional strategies (7, 15–17). For example, the addition of functional additives, such as functional amino acids (18), nucleotides (19), probiotics (7) as well as curcumin (15–17, 20) in the diet can promote intestinal improve the intestinal antioxidant capacity and immunity, and improve gut health of IUGR piglets.

Curcumin [1,7-bis(4-hydroxy-3-methoxyphenyl)-1,6-heptadiene-3,5-dione], as a natural lipophilic polyphenol derived from the rhizome of *Curcuma longa*, has been used for centuries in traditional Asian medicine and food additives (21–23). Nowadays, curcumin has received considerable attention in animal husbandry because of its diverse pharmacological activities including antioxidant (16), anti-microbial (24), and anti-inflammatory properties (25). Research evidence showed that curcumin supplementation can effectively improve the antioxidant capacity, improve digestion and absorption and promote the development and repair of the damaged intestinal tract, and enhance the growth performance of IUGR piglets (15–17, 20). However, the application of curcumin in animal production is limited due to its low bioavailability caused by poor solubility, chemical instability, and rapid degradation. A good understanding of the characteristics of curcumin is the precondition to improve its application. The purpose of this paper is to review the physical and chemical properties of curcumin and its metabolites and its nutritional effects on intestinal health from the aspect of protecting IUGR piglets from oxidative damage. This review provides a theoretical basis for the application of curcumin in animals and humans with IUGR.

## Overview of Curcumin

**FIGURE 1:**
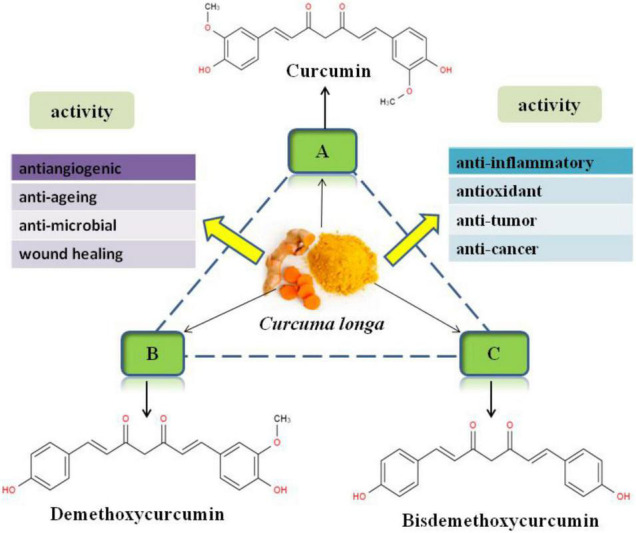
Chemical structures of curcuminoids and their main biological function.

Curcumin is mainly derived from the rhizome of *Curcuma longa* (turmeric), a kind of plant belongs to Zingiberaceae which contains more than 12 active components (26). Commercially, curcumin is one of the main active components in turmeric, which accounted for 77% of active components besides two other related compounds, demethoxycurcumin and bis-demethoxycurcumin (Figure 1) (27). Curcumin is a kind of natural polyphenol that possess a wide spectrum of biological and pharmacological activities, including anti-inflammatory (28–30), antioxidant (31–33), anti-tumor (34, 35), anti-cancer (36, 37), antiangiogenic (38), anti-aging (39), anti-microbial (24), and wound healing (40) activities, which confirmed by *in vitro* and *in vivo* studies. Chemically, curcumin is a bis-α,β-unsaturated β-diketone with two benzene rings that have phenolic hydroxyl and the methoxy, respectively (Figure 1). The molecular formula of curcumin is C_21_H_20_O_6_ with a molecular weight of 368.37 g/mol, and a melting point of 183°C (41).

Curcumin is insoluble in water while it is easily soluble in organic solvents, alkali and extremely acidic solvents (27, 42). It has been reported that under acidic and neutral conditions, curcumin is stable, while under alkaline conditions, curcumin is unstable and easily degrades into other organic substances, including ferulic acid, feruloyl methane, vanillin, vanillic acid, ferulic aldehyde, 4-vinyl guaiacol, *p*-hydroxybenzaldehyde, and *p*-hydroxybenzoic acid, suggesting that pH-dependent stability (27, 43).

The absorption, distribution, metabolism, and excretion of curcumin are critical for its bioavailability. The poor solubility, chemical instability, and rapid degradation have been reported as a cause for the low bioavailability of curcumin (44, 45), which limits its application in animal production. Previous studies have demonstrated that curcumin is poorly absorbed by intestinal cells, rapidly metabolized by the liver, and rapidly eliminated from organism (39, 46, 47), Thus, structural characteristics should be considered to improve its bioavailability and enhance its biological activities. Hence, different strategies were tested to improve its bioavailability, e.g., curcumin nanoparticles, curcumin nanospheres, and emulsion or microsphere preparations of curcumin (48–52). Encapsulation of curcumin into water-soluble proteins or water-insoluble proteins seems to be an effective manner to enhance its antioxidant capacity. Tapal and Tiku et al. (53) reported that the binding of curcumin to soy protein isolate improved its water solubility, stability, and antioxidant activity of curcumin. Moghadam et al. (54) showed that the encapsulation of curcumin by pH-driven method into walnut proteins improved its water solubility, free radical [1,1-diphenyl-2-picrylhydrazyl (DPPH) and 2,2′-azino-bis (3-ethylbenzothiazoline-6-sulfonic) acid (ABTS)] scavenging activity as well as reducing power. Similarly, Mohammadian et al. (55) also reported that complexed curcumin with whey protein nanofibrils could drastically improve DPPH radical scavenging activity and reduce power. Structural modification is another way to improve the antioxidant capacity of curcumin. With the great potential of nanotechnology, modification of curcumin with colloidal nanoparticles has been shown to improve biological activities (56). In this regard, Chen et al. (57) demonstrated that the supplementation of nanobubble curcumin could help mice to overcome physical fatigue by altering the gut microbiota composition. Research by Shaikh et al. (58) reported that structural modification of curcumin to its isoxazole (CI) and pyrazole (CP) showed high reactivity toward a variety of free radicals. However, in recent years, researchers found that the potential biological function of curcumin may not depend on its bioavailability, but may come from its positive impact on gastrointestinal health and function (59). For example, dietary supplementation with curcumin would regulate the intestinal permeability, influence of intestinal flora structure, reduce gastrointestinal inflammation and oxidative stress, and reduce the intestinal pathogens infection (23, 45, 59–61). What else, curcumin’s main metabolites may have stronger pharmacological activity and higher bioavailability than curcumin, which are involved in the biological activities of curcumin (59). However, the biological activities of curcumin’s main metabolites differed among different studies. For example, Luo et al. (62) indicated that compared with curcumin, tetrahydrocurcumin and octahydrocurcumin (two important metabolites of curcumin) can bind to the active site of cytochrome enzyme CYP2E1 to inhibit its activity and simultaneously activate the antioxidant signaling pathway. Zhang et al. (63) showed that tetrahydrocurcumin and octahydroturmeric exerted more effect than curcumin in selectively inhibiting the expression of cyclooxygenase 2 (COX-2) and suppressing nuclear factor-κB (NF-κB) pathways; while, Zhao et al. (29) indicated that curcumin exerted a more potent effect on lipopolysaccharide (LPS)-challenged RAW 264.7 cells compared to that of its three metabolites, tetrahydrocurcumin, hexahydrocurcumin, and octahydroturmeric. Thus, whether the metabolites of curcumin can explain the biological activities is yet to be validated.

## Curcumin Promotes Growth and Intestinal Development of Intrauterine Growth Restriction Pigs

In addition to its anti-inflammatory, antioxidant, immunomodulatory, and other biological functions, curcumin has been reported to promote growth performance and intestinal development of animals. Nowadays, curcumin was widely applied in poultry (64–69), ruminant (70, 71), aquatic animals (72–75), and swine production (76–79).

### Curcumin Promotes Growth Performance of Pigs With Intrauterine Growth Restriction

Intrauterine growth restriction, defined as fetal weight less than the 10^th^ percentile for gestational age, has adverse effects on animal’s growth and development (17, 80). In actual production, IUGR occurs in 15–20% of newborn piglets, which causes considerable economic losses in swine production (81). IUGR has a significant negative effect on the growth and health status of piglets, and IUGR pig neonates manifest retard growth, weak immunity, and poor feed efficiency (1, 82). Xiong et al. (81) showed that compared to normal-birth-weight (NBW) pigs, IUGR pigs had lower initial (1.86 kg vs. 0.96 kg), weaned (6.57 kg vs. 3.66 kg), and final body weight (105.40 kg vs. 81.71 kg); Niu et al. (17) showed that the body weight of IUGR piglets were lower than those of the NBW piglets at 0, 7, 14, and 26 days of age. In brief, IUGR have an adverse effect on growth performance of pigs. Previous studies reported that these conditions can be attenuated by the supplementation of curcumin in the diets of IUGR piglets because of its affordability and safety, with no known toxic side effects (16, 17, 20, 78). Wang et al. (83) showed that the total weight gain and total feed intake of piglets with IUGR were significantly lower than that of NBW piglets in a 24-day experiment, while IUGR piglets fed a diet containing 400 mg/kg curcumin significantly increased the total weight gain and total feed intake. Similarly, Niu et al. (16, 17) reported that dietary curcumin supplementation (400 mg/kg diet) significantly improved the body weight gain and feed intake of IUGR piglets compared with IUGR piglets fed only basal diet. These studies demonstrated that curcumin can promote the growth of piglets with IUGR. In contrast, the results from Zhang et al. showed that dietary supplementation with 200 mg/kg curcumin did not affect the body weight of IUGR piglets on day 0, 26, 56, and 115 of the experimental period when compared with IUGR piglets without curcumin supplementation; and it also recorded a lower ADFI of IUGR piglets fed a diet containing curcumin from day 56 to day 115, while observed improvement in the redox status and meat quality of leg muscles (78). The difference among these studies may be related to the different doses of curcumin used. Since the bioavailability of curcumin is very low due to its poor solubility (44, 45), high doses are required to achieve detectable levels in serum, which can exert its biological function.

### Curcumin Promotes Intestinal Development of Pigs With Intrauterine Growth Restriction

The intestinal tract is not only the direct place for nutrient digestion and absorption but also provided an important barrier to protect the body from antigens, toxins, and pathogens and maintain the stability of the internal environment (8, 9). Therefore, well-developed and healthy intestines are linked with the overall health status of animals. IUGR is a common problem in the pig industry, and a change in intestinal morphology between IUGR piglets and NBW piglets was observed (84). Several studies have reported that IUGR piglets had a lower digestive and absorptive function and an impaired intestinal barrier function (5, 6, 84). It showed that IUGR piglets had a decreased intestinal length and weight, shorty VH, increased apoptosis of intestinal crypt cells as well as reduced activity of brush border enzymes, which leads to an increase in the occurrence of diarrhea and high morbidity and mortality after birth (5, 14).

As a natural polyphenol with a variety of biological activities, curcumin can promote intestinal development and health (67, 76). For example, adding 300 mg/kg or 400 mg/kg curcumin to diet can significantly increase villus height to crypt depth ratio (VCR), improve the morphology of ileum epithelial mucosa, and repair the intestinal injury in *Escherichia coli* (*E. coli*) induced intestinal injury piglets model (76). Curcumin can also promote the intestinal development of animals with IUGR including piglets. The intestinal VH, CD, and VCR are commonly used to reflect intestinal development and function (85). Wang et al. (83) showed that IUGR piglets have a poor intestinal morphology, which manifested by a decreased VH and VCR, and increased CD in duodenum, jejunum, and ileum; while, dietary curcumin supplementation (400 mg/kg diet) significantly increased the VH and VCR, which indicated that curcumin has a positive protective effect on improving intestinal morphological damage caused by IUGR in piglets. Similarly, Yan et al. (15) indicated that curcumin can alleviate the jejunum injury in IUGR piglets by increasing the antioxidant capacity.

Disaccharidase (lactase, maltase, and sucrase) activities are important indicators of intestinal functional development (86, 87). In a rabbit model, the authors found that both lactase and maltase activities were depressed in IUGR fetuses compared with NBW ones (86). Likewise, the maltase and lactase in the jejunum and the maltase and sucrase in the ileum were significantly decreased when piglets suffered from IUGR (83). It means that IUGR affects the secretion and activity of intestinal digestive enzymes and hinders the digestion and utilization of nutrients in weaned piglets. Curcumin can reverse this adverse effect caused by IUGR which was indicated by Wang et al. (83) who reported that diet supplemented with 400 mg/kg curcumin significantly improved the ileum lactase activity of IUGR weaned piglets.

## Curcumin and Intestinal Antioxidant Function of Intrauterine Growth Restriction Pigs

Curcumin is a polyphenol, characterized by the inclusion of two aromatic rings, and its phenolic hydrogens are responsible for its ability to react with reactive species and are believed to impart antioxidant activity to the molecule (88). So far, data from *in vivo* and *in vitro* studies have shown the antioxidant activity of curcumin in different pathological conditions through different pathways (89, 90). The antioxidant activity of curcumin mainly from two aspects: one is curcumin as a free radical scavenger (91, 92); and the other is curcumin as inducers of antioxidant signaling pathways in cells, by enhancing the activity of antioxidant enzymes, such as superoxide dismutase (SOD), catalase (CAT), glutathione peroxidase (GSH-Px), and phase II metabolizing enzymes, heme oxygenase (HO-1) and quinone oxidoreductase (NQO1) (33, 67, 93, 94). Hence, curcumin may be a beneficial antioxidant to prevent oxidative damage.

### The Ability of Curcumin to Scavenge Free Radicals

High reactive oxygen species (ROS) and reactive nitrogen species (RNS) are devastating for cells, and therefore free radical scavenging is important for preventing some diseases (9, 89). The antioxidant activity of a substance is evaluated by the ability to scavenge nitric oxide (NO), DPPH, ABTS, superoxide radical (O^2–^), hydrogen peroxide (H_2_O_2_) (9, 95–97). Previous studies have demonstrated that curcumin has a strong free radical scavenging activity, thereby protecting against oxidative damage (94, 98). For example, Borra et al. (98) showed that curcumin could efficiently scavenge DPPH, H_2_O_2_, NO, superoxide anion in a dose-dependent manner. Ferric-reducing antioxidant power (FRAP) and reducing power assay represent their ability to reduce the ferric (Fe^3+^) form to the ferrous (Fe^2+^) form (9, 99). Curcumin also could efficiently scavenge the peroxy radicals, which can induce hemolysis in erythrocytes and inhibit the erythrocyte membrane lipid peroxidation (94). Barzegar et al. (100) showed that curcumin exhibited scavenging intracellular smaller oxidative molecules including H_2_O_2_, HO^–^, ROO^–^, and can readily transfer electrons or easily donate H-atom from two phenolic sites to scavenge free radicals. These studies indicated that curcumin can be used as an effective antioxidant for ROS protection within the polar cytoplasm due to its superb intracellular ROS scavenging activity.

### *In vivo* and *in vitro* Antioxidant Activity of Curcumin

Curcumin is a natural phenolic compound with impressive antioxidant properties which has gained increasing attention owing to its beneficial health properties (31, 101). Previous studies showed that curcumin can relieve oxidative stress caused by many unfavorable factors (102–104). *In vitro* and *in vivo* studies showed that curcumin is an important inducer of nuclear factor erythroid 2-related factor 2 (Nrf2)-mediated antioxidant signaling pathways (15, 105). Nrf2 is the main regulator of mammalian cell redox response and plays a vital role in maintaining cellular homeostasis (106, 107). Under normal physiological conditions, kelch-like ECH-associated protein-1 (Keap1) binds to Nrf2 in the cytoplasm and facilitates Nrf2 ubiquitination which can prevent Nrf2 translocation into the nucleus (107). But under oxidative stress conditions, Nrf2 was isolated from Keap1 and transferred to the nucleus, and bound with antioxidant response elements (ARE) to activate the expression of its downstream antioxidant enzymes (SOD, CAT, and GSH-Px), and phase II metabolic enzymes (HO-1 and NQO1) to protect cells from oxidative damage (106–109). For example, Wu et al. (110) reported that the chicken fibroblast cells suffered from heat stress stimulate ROS and malondialdehyde (MDA) production, and it decreased the antioxidant enzymes including CAT, SOD, and GSH-Px; curcumin administration reversed these heat stress-induced oxidative damage by activating Nrf2 signaling pathway. Similarly, Li et al. (111) reported that dietary 300 mg/kg diet curcumin supplementation to broilers alleviates aflatoxin B1 induced liver oxidative stress by activating the Nrf2 pathway. The *in vitro* and *in vivo* antioxidant effects of curcumin are summarized in Tables 1, 2, respectively. All these studies revealed that curcumin plays an important role in relieving oxidative stress by improving antioxidant activities.

**TABLE 1:** Summary of the *in vitro* studies investigating the antioxidant effect of curcumin.

Cell lines	Injure model	Doses	Outcomes	References
RAW264.7 cells	Hydrogen peroxide- induced oxidative injure	5, 10, 20 μM	Low- and middle-dose of curcumin decreased MDA and ROS levels; increased activity of CAT, SOD and GSH-Px; upregulated Nrf2 and HO-1 expression	(33)
Bovine fetal hepatocyte-derived cell line (BFH12)	Aflatoxin B1-induced hepatic toxicity	2.5, 5, 10 μM	Reduced the MDA content, increased the NQO1 enzymatic activity	(70)
Porcine intestinal epithelial cells (IPEC-J2)	Hydrogen peroxide- induced oxidative stress	10 μM	Reduced MDA and ROS production, increased the expression of Cu/Zn-SOD, Mn-SOD, GPX-1 and GPX-4	(93)
Bovine Mammary Epithelial Cells	Lipopolysaccharide – induced oxidative stress	10 μM	Decreased production of ROS and MDA; increased the activities of T-SOD, T-AOC and GSH; increased the levels of Nrf2 and HO-1 and NQO1	(105)
Primary spinal cord astrocytes	Hydrogen peroxide- induced oxidative injure	10 μM	Decreased the level of intracellular ROS, and inhibited oxidative stress *via* the Nrf2/ARE pathway	(108)
Chicken embryonic fibroblasts cells	Heat-induced oxidative stress	5 μM – 40 μM,	Decreased ROS and MDA content; increased antioxidant enzymes and Nrf2 expression	(110)
Human trophoblast HTR8/SVneo cells	H_2_O_2_-induced oxidative stress	2.5 or 5 μM	Reduced ROS accumulation, upregulated the activities of the antioxidant enzymes CAT and GSH-Px, increased antioxidant transcription factor Nrf2	(112)
SH-SY5Y cells	Copper-induced neurotoxicity	5 μM	Decreased the production of ROS and MDA; increased the activities of SOD and CAT; up-regulated pro-caspase 3, pro-caspase 9, and downregulated the Bax/Bcl-2 ratio	(113)
Leydig cells	Zearalenone-induced oxidative stress	20 μM	Reduced MDA content; increased the GSH content and the activities of GSH-Px; increased nuclear Nrf2 and HO-1 protein expression	(114)
Human retinal pigment epithelium cell lines (ARPE-19)	Hydrogen peroxide- induced oxidative stress	15 μM	Reduced ROS production and increased HO-1 expression	(115)
Bone marrow mesenchymal stem cells (BMSCs)	Hydrogen peroxide- induced oxidative stress	1, 5, 10 or 20 μM	Curcumin pretreatment can inhibit reactive oxygen species accumulation in BMSCs	(116)
Bone marrow mesenchymal stem cells (BMSCs)	Hypoxia and reoxygenation triggered injury	1, 5, 10 or 20 μM	Curcumin pretreatment prevented hypoxia and reoxygenation-induced mitochondrial dysfunction through suppressing reactive oxygen species accumulation	(117)
Porcine granulosa cells	Zearalenone -induced oxidative stress	20 μM	Pre-treated with curcumin decreased the ROS production, and increased the expression of SOD1 and CAT	(118)
Tilapia hepatocytes	Hydrogen peroxide- induced oxidative injure	5, 10, 20, 40 μM	Reduced MDA levels, and increased SOD activity; upregulate the Nrf2-Keap1 signaling pathway at the transcriptional level	(119)
Min-6 mouse pancreatic beta cells	High glucose – induced oxidative stress	10 μM	Decreased MDA and ROS levels; increased SOD activity	(120)
Porcine TM cells	Hydrogen peroxide- induced oxidative injure	1–20 μM	Curcumin treatment at concentrations between 1 and 20 μM reduced the production of intracellular ROS	(121)
INS-1 cells	High glucose/palmitate- induced cell damage	20 μM	Reduced the production of ROS Increased SOD and CAT activity	(122)
Human hepatocyte L02 cells	Quinocetone-induced hepatic toxicity	2.5, 5 μM	Attenuated ROS formation; increased SOD activity and GSH level	(123)
Human intestinal epithelial cells (Caco2)	Hydrogen peroxide- induced oxidative injure	5, 20, 80 μM	Decreased MDA release; increased SOD activity; increased HO-1 expression	(124)
SH-SY5Y cells	Paraquat-induced cell death	5 μM	Curcumin reduced ROS levels and increased expression of the antioxidant genes, SOD and GSH-Px	(125)

*ARE, antioxidant response elements; CAT, catalase; GSH, glutathione; GSH-Px, glutathion peroxidase; HO-1, heme oxygenase; Keap1, kelch-like ECH-associated protein 1; MDA, malonaldehyde; NQO1, quinone oxidoreductase; Nrf2, nuclear factor erythroid 2-related factor 2; ROS, reactive oxygen species; SOD, superoxide dismutase; T-AOC, total antioxidant capacity.*

**TABLE 2:** Summary of the *in vivo* studies investigating the antioxidant effect of curcumin.

Animals	Damaged model	Doses	Outcomes	References
Pigs	Intrauterine growth retardation	200 mg/kg	Increased the gene expression of Nrf2, GCLC, SOD1, GCLM and NQO1, and the protein expression of Nrf2 and NQO1	(15)
Pigs	Intrauterine growth retardation	400 mg/kg	Reduced the levels of MDA and H_2_O_2_; improved serum and liver antioxidant enzymes as well as up-regulated Nrf2 and HO-1 expression	(17)
Pigs	Intrauterine growth retardation	400 mg/kg	Reduced PC, 8-OHdG, increased T-AOC, CAT, SOD, Nrf2, NQO1 expression	(20)
Pigs	Intrauterine growth retardation	200 mg/kg	Increased mRNA expressions of GSH-ST, HO-1 and CAT, increased NQO1 protein expression of leg muscles	(78)
Pigs	Diquat -induced oxidative stress	200 mg/kg	Reduced the MDA level, and increased the SOD, CAT activity in the intestinal mucosa	(93)
Rats	Intestinal ischemia reperfusion	100 mg/kg	Decreased the MDA levels, and increased of SOD and GSH-Px enzyme activities	(60)
Rats	Dimethylnitrosamine-induced liver injury	200 mg/kg	Enhanced antioxidant transcription and ARE-binding of Nrf2; increased HO-1 protein expression as well as activity in rat liver	(102)
Rats	Lipopolysaccharide/diclofenac-induced liver injury	200 mg/kg/d	Decreased the MDA levels; increased GSH content and SOD enzyme activities; increased expression of HO-1	(103)
Rats	Intestinal ischemia reperfusion	200 mg/kg	Decreased the MDA levels, and increased SOD enzyme activities	(126)
Rats	Renal ischemia reperfusion	15 mg/kg, 30 mg/kg, 60 mg/kg	Decreased MDA; increased the level of SOD, CAT, GSH-Px, GSH	(127)
Rats	Ochratoxin A-induced Hepatotoxicity	100 mg/kg	antioxidant enzymes SOD, CAT and GSH-Px increased; MDA level decreased	(128)
Rats	Streptozoticin -induced diabetic	100 mg/kg/d	The activity of SOD increased and the amount of MDA reduced; the expression of NQO1 and Nrf2 was increased	(129)
Rats	Intrauterine growth retardation	400 mg/kg	Decreased the MDA, PC and 8-OHDG contents, improved the hepatic glutathione redox cycle	(130)
Rats	Aluminum chloride-induced oxidative stress	10 mg/kg BW	Decreased the MDA levels, and increased SOD and CAT activities in liver tissue	(131)
Mice	arsenic-induced hepatotoxicity and oxidative injuries	200 mg/kg	Decreased hepatic MDA level, increased hepatic GSH level, and up-regulated Nrf2 protein, NQO1 and HO-1 expression	(31)
Mice	Cadmium-induced histopathological damages	100 mg/kg	Increased serum CAT, SOD, and GSH-Px activities; decreased the serum MDA and H_2_O_2_ level	(132)
Mice	Cadmium induced lung oxidative stress	100 mg/kg	Decreased MDA levels; increased CAT, GSH-Px,SOD activities	(133)
Mice	Ethanol-induced oxidative stress	50 mg/kg	Reduced ROS and lipid peroxidation (LPO) generation, and increased Nrf2/HO-1 expression in the experimental mice brains	(134)
Ducks	Ochratoxin A induced liver oxidative injury	400 mg/kg	Increased liver CAT activity	(24)
Ducks	Aflatoxin B1-induced intestinal injure	500 mg/kg	Enhanced the activities of SOD, GSH-Px, GSH-ST; decreased the concentrations of MDA in the ileum	(67)
Ducks	Ochratoxin A–induced intestinal injure	400 mg/kg	Decreased the concentrations of MDA; increased the activity of GSH-Px in the jejunal mucosa	(135)
Broilers	Aflatoxin B1-induced liver injury	300 mg/kg	Inhibited the generation of ROS, MDA and 8-OHdG; increased the activities of GSH, SOD and CAT; increased the expression of Nrf2 and HO-1	(111)
Broilers	Aflatoxin B1-induced liver injury	300 mg/kg	Decreased the content of MDA and the level of ROS; increased the contents of GSH and activities of SOD and CAT	(136)
Broilers	Aflatoxin B1-induced liver injury	450 mg/kg	Decreased the MDA levels, and increased GSH-Px and SOD activity; up-regulated Nrf2 protein expression	(137)
Broilers	Aflatoxin B1-induced liver injury	300 mg/kg	Improved Nrf2 expression, and Enhanced phase-II metabolizing enzymes expressions and activity	(138)
Laying hens	Heat-induced oxidative stress	100 to 300 mg/kg	Decreased the MDA levels; increased T-AOC, CAT, SOD and GSH-Px activities	(139)

*8-OHdG, 8-hydroxy-2′-deoxyguanosine; ARE, antioxidant response elements; CAT, catalase; GCLC, glutamate-cysteine ligase catalytic subunit; GCLM, glutamate-cysteine ligase modifier subunit; GSH, glutathione; GSH-Px, glutathion peroxidase; GSH-ST, glutathione S-transferase; HO-1, heme oxygenase; H_2_O_2_, hydrogen peroxide; Keap1, kelch-like ECH-associated protein 1; MDA, malonaldehyde; NQO1, quinone oxidoreductase; Nrf2, nuclear factor erythroid 2-related factor 2; PC, protein carbonyl; ROS, reactive oxygen species; SOD, superoxide dismutase; T-AOC, total antioxidant capacity.*

### Curcumin Alleviates Intestinal Oxidative Stress in Intrauterine Growth Restriction Pigs

Oxidative stress, recognized as a state of imbalance between the production of free radicals and antioxidant defenses, plays a crucial role in the development of numerous human and animal diseases (107, 140, 141). In cells, free radicals are unstable compounds that readily bind to oxygen to become reactive species such as ROS and RNS, causing cytotoxic effects (85, 142). Free radicals are a double-edged sword, on the one hand, physiological levels of ROS and RNS are required for some enzymatic, cell signaling, and cellular adaptive responses; while on the other hand the excessive production of free radicals, which in turn, induce oxidative damage to cellular biomolecules, including proteins, lipids, and nucleic acids (128, 143). Oxidative stress is associated with IUGR (86, 144, 145). Previous studies have revealed that IUGR offsprings tend to have increased ROS, 8-OHdG, protein carbonyl (PC), MDA, and H_2_O_2_, and decreased levels of antioxidant enzymes (SOD, CAT, GSH-Px), and phase II metabolizing enzymes (HO-1 and NQO1) (15, 17–20, 130, 146). IUGR is associated with intestinal oxidative stress in weaned piglets (15, 20). Substantial evidence has indicated that oxidative stress triggered the onset and development of intestinal diseases as well as implicated in the pathophysiology of IUGR-associated intestinal injury (15, 147, 148). It is believed that oxidative stress is involved in intestinal barrier dysfunction and various digestive tract diseases (107, 149). At present many natural oxidation products have been used to alleviate oxidative stress in IUGR pigs (146, 148), in which curcumin has been mentioned as a remedy. Wang et al. (20) showed that IUGR stimulated jejunum PC and 8-OHdG, and ileum PC, MDA, and H_2_O_2_ production, and it decreased the total antioxidant capacity (T-AOC), CAT activity, and glutathione (GSH) content in the jejunum, and CAT activity in the ileum, which suggested that IUGR caused oxidative stress in the intestinal tract. The authors further reported that administration of curcumin at a dose of 400 mg/kg reversed IUGR associated intestinal damage by activating the Nrf2 signaling pathway and stimulating antioxidant enzymes secretion (SOD and CAT), and phase II metabolic enzyme, NQO1 expression. Similarly, Yan et al. (15) showed that the IUGR growing pigs fed a diet containing 200 mg/kg curcumin had significantly lower MDA content and higher total SOD activity in the jejunum, and upregulated Nrf2, NQO1, and SOD expression. These studies suggested that curcumin can alleviate intestinal oxidative stress caused by IUGR and improve intestinal antioxidant status through activating Nrf2/ARE signaling pathway.

## Conclusion

**FIGURE 2:**
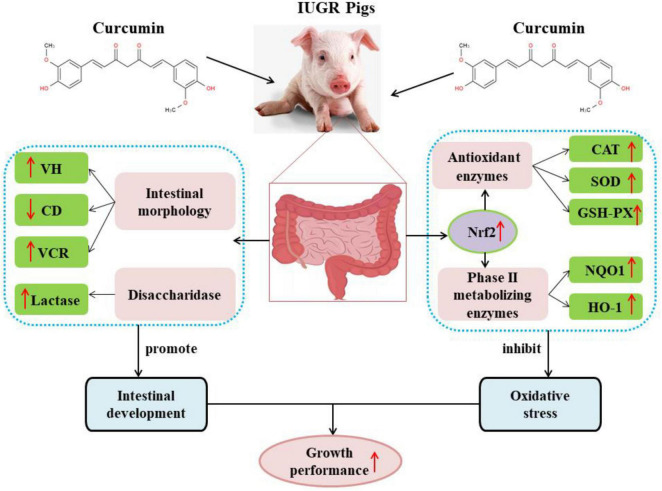
Curcumin improved the growth performance of pigs with IUGR by improving intestinal development and alleviating intestinal oxidative stress. CAT, catalase; CD, crypt depth; GSH-Px, glutathion peroxidase; HO-1, heme oxygenase; NQO1, quinone oxidoreductase; Nrf2, nuclear factor erythroid 2-related factor 2; SOD, superoxide dismutase. VCR, the ratio of villus height to crypt depth; VH, villus height.

In conclusion, curcumin has a good antioxidant capacity with a strong free radical scavenging activity and can effectively improve intestinal development and alleviate intestinal oxidative stress caused by IUGR, thereby improving the growth performance and health status of pigs with IUGR (Figure 2), however, the mechanism of curcumin in relieving intestinal oxidative stress and intestinal dysplasia in IUGR piglets is yet to be investigated. Curcumin exhibited low bioavailability due to poor solubility, chemical instability and rapid degradation, and those will limited its application in animal production. Therefore, further studies should focus on how to improve the bioavailability of curcumin to enhance biological activities.

## Author Contributions

XT, KX, and XW advocated writing this review and reviewed. XT collected literature and wrote the manuscript. TW revised the manuscript. All authors contributed to the article and approved the submitted version.

## Conflict of Interest

The authors declare that the research was conducted in the absence of any commercial or financial relationships that could be construed as a potential conflict of interest.

## Publisher’s Note

All claims expressed in this article are solely those of the authors and do not necessarily represent those of their affiliated organizations, or those of the publisher, the editors and the reviewers. Any product that may be evaluated in this article, or claim that may be made by its manufacturer, is not guaranteed or endorsed by the publisher.
